# American Medical Association

**Published:** 1857-06

**Authors:** 


### EDITORIAL.

*American Medical Association.*

The tenth annual meeting of this important organization was held on the 5th, 6th, and 7th, days of May, in the city of Nashville, Tennessee. Owing to the length of time required to reach the place of meeting from the North-West and North, the number of delegates in attendance was not as large as at former meetings of the Association. We had intended to give a full record of the Proceedings, in this number of the Journal, but the reports furnished through the Nashville papers are too incomplete for our purpose, and we shall, therefore, wait until an official copy is received. The following are the officers chosen for the present year, viz. :—

Dr. Paul F. Eve, of Tennessee, *President.*

Drs. R. J. Breckenridge, of Kentucky; D. M. Reese, of New York; W. H. Byford, of Indiana; and H. F. Campbell, of Georgia, *Vice-President.*

Drs. R. C. Foster, of Nashville, and A. J. Semmes, of Washington City, *Secretaries.*

The place selected for holding the next Annual Meeting is Washington, D. C. We clip the following from the *Republican Banner*, a Nashville paper :—

*American Medical Association.*—This distinguished body adjourned *sine die* yesterday at 2 o'clock. The Association transacted a large amount of business for the time occupied, very little time being occupied in discussion. The subject of Medical Education aroused some feeling yesterday morning, and a very able debate grew out of it.

The occasion has been one of great pleasure to our citizens, and we trust no less so to the members of the Association.

The festivities were concluded last night by a grand ball and supper at the Capitol, at which was assembled an array of beauty and fashion which could scarcely be eclipsed in brilliancy anywhere.

The members generally leave for their homes to-day. We wish them one and all a safe return to their homes and families.

P.S.—We have just received extra sheets of the *Nashville Journal*, with the Proceedings in full; for which the editors will accept our thanks.

